# The Prevalence and Correlates of Child Sexual Offending Behaviours and Attitudes Among Men in Australia, the United Kingdom, and the United States: Study Methodology

**DOI:** 10.1177/08862605251403613

**Published:** 2026-03-03

**Authors:** Tyson Whitten, Michael Salter, Delanie Woodlock

**Affiliations:** 1 University of New South Wales, Sydney, Australia

**Keywords:** sexual abuse, child maltreatment, offenders/perpetrators, research methodology/measurement

## Abstract

This paper describes the methodology for an online survey of the prevalence and factors associated with interest and behaviours towards children among men aged 18 years or older from the general Australian, U.K., and U.S. adult male populations. The study collected data on demographic characteristics, health issues, social support, childhood adversity, and patterns of technology and internet use, as well as attitudes and behaviours relating to online and offline child sexual exploitation and abuse (CSEA). Surveys were administered through Prime Panels using census-matched quotas. Data were subsequently weighted via iterative proportional fitting. Of the 7,343 people who consented to participate in the survey, 4,918 were retained (Australia = 1,939; United Kingdom = 1,506; United States = 1,473). The demographic characteristics for the weighted samples were comparable to the Australian, U.K., and U.S. male census benchmarks. The proportion of men who engaged in online CSEA or had hebephiliac interest was also comparable to the pooled prevalence obtained from 11 other non-clinical, community samples. Despite this study’s strengths, including its international scope and broad data collection, the study is limited by the potential for selection and social desirability bias, as well as the implications of not distinguishing the ages of participants in consensual sexual activities. Future research directions include expanding the study to non-English and low-income countries and integrating longitudinal and qualitative methodologies.

## Introduction

Preventing child sexual exploitation and abuse (CSEA) requires precise information on the prevalence of undetected offenders, their characteristics, and how they operate online. However, current research is largely limited to data obtained from forensic or convenience samples. Studies using large, representative samples are scarce, mostly confined to European populations, and lack data on offenders’ regular online activities, social supports, and various adversity indicators that could improve detection efforts (Savoie et al., 2021). Additionally, there has been no international comparison of the characteristics and online behaviours of undetected offenders in the general population. This omission implies an assumed uniformity in offender profiles across countries, ignoring how differences in online regulation and surveillance might affect offending patterns. Ultimately, policy makers and stakeholders need dependable, country-specific estimates derived from the general population to develop effective strategies at scale for identifying and preventing CSEA in the community.

This article describes the methodology for an online survey of the prevalence and factors associated with sexual interest and behaviours towards children among men aged 18 years or older from the general Australian, U.K., and U.S. male populations. The study extends earlier investigations into CSEA within community samples by incorporating a broader scope. Unlike prior research that concentrated on psychological correlates and risk factors, this study expanded the data collection to include demographic characteristics, health issues, social support, childhood adversity, and patterns of technology and internet use, as well as attitudes and behaviours relating to online and offline CSEA.

This study focuses on men due to their majority status among CSEA offenders, recognising that female CSEA offenders, while exhibiting some overlapping patterns, significantly differ in their risk factors, circumstances, motivations, and tactics (Burgess-Proctor et al., 2017). Importantly, the study broadly defined children as anyone under the age 18 years, which is consistent with the UN Convention on the Rights of the Child’s international violence standards. We recognise that the legal age of consent varies globally, typically between age 16 and 18 years. However, transmitting, accessing, and soliciting online sexual material of someone under the age of 18 years is illegal in all countries covered in this study (e.g. Australia: Criminal Code Act 1995 *(Cth) s. 473.1;* United States: *18 U.S.C. § 2252*; United Kingdom: Protection of Children Act 1978, *c. 37, s.1*). Nonetheless, this study does not specify the exact ages of the involved parties at the time of these behaviours other than if they were above or below age 18 years.

## Methodology

The survey was developed by the research team and reviewed by a project advisory group, which included representatives from law enforcement, financial intelligence units, government departments, and mental health support services. Funding was provided by Westpac’s “Safer Children, Safer Communities” programme as part of a collaborative research project between academia and civil society. Ethical approval was provided by the University of New South Wales (HC220317). The survey was uploaded to Qualtrics and administered via an online panel, given that such platforms are well suited for sensitive research topics where people would traditionally be reluctant to participate or respond honestly (Burkill et al., 2016; Gnambs & Kaspar, 2015). Dynata (https://www.dynata.com), the online research panel company initially commissioned to administer the survey, declined to do so upon reviewing the questionnaire and deeming the subject matter “immoral.”

CloudResearch (https://www.cloudresearch.com), an online research platform with access to over 100 million participants globally, subsequently agreed to recruit and administer the online survey. The survey was conducted using Prime Panels, which aggregates numerous market research platforms, each with its own opt-in participant pool profiled on hundreds of variables. Targeted invitations were sent to participants based on their demographic profiles. Participants were paid an undisclosed amount at the discretion of each market research platform. Data collection commenced with a small pilot (*n* = 150) to verify functionality before the full data was collected.

### Sampling Design and Participants

From November to December 2022, survey invitations were sent to quota-based samples of men aged 18 years or over, living in Australia, the United Kingdom, and the United States. The target sample size was 1,500 eligible participants per country. Given Prime Panels aggregates survey participants from multiple platforms, the total number of people invited to participate or who accessed the study could not be ascertained. Assuming that the response distribution of child sex offending among adult men is 5%, the 95% confidence interval for the margin of error is ±1.10%. Quota categories were limited to the pre-survey participant demographic data available to CloudResearch, which include age, ethnicity (only available for U.S. participants), residential region, annual household income before tax, and highest educational attainment. “Best effort” target quotas were derived from each country’s 2021 census of the adult male population (Australian Bureau of Statistics [ABS], 2021; Office for National Statistics [ONS], 2021; United States Census Bureau, 2021) and are presented below in Table 1.

**Table 1.** Targeted (%) Demographic Quotas for Eligible Participants.

Demographics	Australia	United Kingdom	United States
Age (years)	18–24 (11%); 25–34 (19%); 35–44 (18%); 45–54 (16%); 55–64 (15%); 65 or older (22%).	18–24 (11%); 25–34 (17%); 35–44 (16%); 45–54 (17%); 55–64 (16%); 65 or older (24%).	18–24 (12%), 25–34 (18%), 35–44 (16%), 45–54 (16%), 55–64 (17%), 65 or older (21%).
Ethnicity	–	–	Black (13%); Hispanic (16%), White (59%); Other (12%).
Region	Sydney (21%); NSW excluding Sydney (11%); Melbourne (20%); VIC excluding Melbourne (6%); Brisbane (10%); QLD excluding Brisbane (10%); Perth (8%); WA excluding Perth (2%); Adelaide (5%); SA excluding Adelaide (2%); Hobart (1%); TAS excluding Hobart (1%); Darwin (1%); ACT and NT excluding Darwin (1%).	North East (4%); North West (11%); Yorkshire and The Humber (8%); East Midlands (7%); West Midlands (9%); East (9%); London (13%); South East (14%); South West (8%); Northern Ireland (3%); Scotland (8%); Wales (5%).	Midwest (21%); Northeast (17%); South (38%); West (24%).
Household income	Less than Aud$55,000 (29%); Aud$55,000–$99,999 (27%); Aud$100,000 or more (44%).	Less than £30,000 (30%); £30,000–£59,999 (39%); £60,000 or more (31%).	Less than US$35,000 (25%); US$35,000–$74,999 (27%); US$75,000 or more (48%).
Education	Did not complete grade 12 (21%); completed grade 12 OR TAFE certificate/diploma (47%); Bachelor’s degree or higher (32%).	Below O-level/GCSE (23%); completed O-levels/GCSEs or equivalent OR A-levels or equivalent OR further qualification between high school and university (45%); Bachelor’s degree or higher (32%).	Some high school or less (10%); High school graduate OR post high-school vocational training (47%); Associate’s degree or higher (42%).

Prospective participants were informed that the survey would take around 15 to 20 min to complete, and that they would receive compensation upon its completion (the exact value was undisclosed to the research team). Upon opening the survey, participants were presented with detailed information regarding its purpose, contents, ethical approvals, risks and benefits, information regarding the research team, assurances of anonymity, and the contact details of local resources for those who may experience distress (also presented at the end of the survey). Participants could only proceed to the survey if they indicated that they had (a) read the participation information, (b) understood their right to withdraw at any time without prejudice, (c) consented to participating in the study, and (d) consented to the use of their information for the purposes of this research.

Each participant was assigned a unique random ID, and no personally identifiable information was recorded. Surveys were hosted on researcher-owned Qualtrics accounts, ensuring that CloudResearch did not access or store responses. Data were password protected and transmitted using transport layer security encryption.

### Measures

The survey was designed according to the recommendations of academic experts in child sex abuse, practitioners, people with lived experiences as a victim-survivor of child sex abuse, and the project advisory group. The survey was also administered to a small convenience sample of Australian men who provided feedback regarding its length and coherence. The final survey measured eight broad domains, designated (a) demographic factors, (b) internet use, (c) physical and mental health, (d) social adversity, (e) attitudes towards child sex abuse, (f) pornography viewership, (g) child sexual exploitation, and (h) sexual interest towards children. The key outcomes for this study, men’s sexual interest and behaviours towards children, were adapted from measures used in prior research on child sex offending. A summary of the survey measures is presented in Table 2, and a detailed outline of all survey items and response categories are available in Supplemental Table S1.

**Table 2.** Summary of Survey Measures by Domain.

Domain	Measures
Demographic factors	• Sex recorded at birth, gender identity, gender of sexual partners, sexual orientation, age, residential location, marital status, educational attainment, employment status over the last 3 months, annual household income before taxes, cultural background, country of birth, number of children living in household, work involves contact with children
Internet use	• Average hours per day online (2 items) • Frequency of routine online activities (13 items; Brewer et al., 2020) • Social media platforms currently used (13 items) • Anonymity and privacy software currently used (10 items) • Cryptocurrency ownership and use (2 items)
Physical and mental health	• Patient Health Questionnaire-4 (4 items; Löwe et al., 2010) • National Institute on Drug Abuse Quick Screen v1.0 (4 items; Zgierska et al., 2014) • Brief disability questionnaire (3 items; Von Korff et al., 1996)
Social adversity	• Multidimensional Scale of Perceived Social Supports (12 items; Zimet et al., 1990) • Adverse Childhood Experiences scale (10 items; Felitti et al., 1998)
Attitudes towards child sex abuse	• Modified version of the Child Sexual Abuse Myth Scale (25 items; Collings 1997) • Beliefs about people who view online sexual content of children (open response)
Pornography viewership	• Accidental and deliberate viewership of pornography involving violence or animals (4 items; Seto et al., 2015) • Purchases of sexual services online (9 items)
Child sexual exploitation and abuse	• Peer engagement in child sexual abuse (3 items; Seto et al., 2015) • Accidental and deliberate viewership of online CSEA (2 items; Seto et al., 2015) • Online and offline sexual contact with children (4 items; Ó Ciardha et al., 2022; Wurtele et al., 2014) • Proclivity to have sexual contact with children (5 items; Ó Ciardha et al., 2022; Wurtele et al., 2014)
Sexual interest towards children	• Ages of sexual attraction (3 items; Ó Ciardha et al., 2022) • Concerns about interest towards children (1 item)

### Analytical Strategy

First presented are the sample participation, completion, and retention rates. Next, the representativeness of the study was demonstrated by comparing the demographic characteristics of the Australian, U.K., and U.S. samples with those of the male population according to the respective 2021 census, before and after applying sample weights. Benchmark weights were based on census data of Australian males aged 15 years and over (ABS, 2021),^1^ U.K. males aged 16 years and over (ONS, 2021)^2^, and U.S. males aged 18 years or over (U.S Census Bureau, 2021).^3^ Iterative proportional fitting was conducted to improve the generalisability of the sample by calibrating the weight of each participant until the sample distribution was concordant with the population distribution (Speed, 2005) according to age, annual household income, race/cultural background, educational attainment, marital status, and workforce participation. Weighted scores that exceeded the median weight plus six times the interquartile range were truncated to reduce mean squared errors of the outcome estimates (Battaglia et al., 2009).^4^

To contextualise our study within the findings of the broader research, we conducted a purposive, non-systematic review identifying manuscripts published since 2010 that report the proportion of men in non-clinical community samples who (a) have engaged in online CSEA,^5^ (b) offline sexual contact with children,^6^ (c) are sexually attracted to pubescent children (11–16 years), and (d) are sexually attracted to prepubescent children (under 11 years). Eleven studies were included (marked * in the reference list), most (73%) of which were not representative of the focal male population. There was considerable between-study variation in methodology and definitions of sexual interest and behaviours towards children, as is common in the field (Savoie et al., 2021). Nonetheless, for demonstrative purposes, a proportional meta-analysis was conducted to obtain the pooled prevalence of these studies. Random effect models were used for all analyses due to between-study heterogeneity (Higgins, 2008).

All non-demographic survey questions required participants to provide an answer before moving on to the next question. However, for some items relating to child sex abuse, participants could respond with “unsure.” We summarise the proportion of these responses. We also present the percentage of men who had any concerns about their sexual interest towards children and would like more information and support, as well as a brief overview of the comments provided by participants at the end of the survey.

Finally, the study does not ask participants to specify the ages at which they engaged in offline sexual behaviours with children (i.e. anyone under 18 years of age). Some consensual sexual activities, such as those between a 17- and 19-year-old, may be categorised as criminal and potentially inflate the prevalence of offline child sex offending. Although we cannot accurately establish the proportion of those who engaged in consensual activity, we attempt to provide a crude estimation by identifying what proportion of men who engaged in offline sexual behaviours with children had other correlates of child sex offending (i.e. online child sex offending, sexual interest towards children, hypothetical engagement in child sex offending, and friends with people who sexually offend against children). Among men who had sexual contact with a child during adulthood, we compare those with and without any other correlates of child sex offending by factors typically associated with sexual offending towards children; these being age, household income, children in household, marital status, educational attainment, sexual orientation, viewing bestiality, viewing violent pornography, and childhood history of sexual abuse (Babchishin et al., 2011; Faust et al., 2015; Salter et al., 2023; Whitaker et al., 2008).

### Statistical Analyses

Analyses were conducted using a complex sample design, with standard errors adjusted to account for poststratification weights (Lumley, 2004). Data are summarised as counts and proportions with accompanying 95% Confidence Intervals (95% CI). Proportional meta-analyses with 95% CI and Prediction Intervals (95% PI) were conducted using the Freeman-Tukey transformations. Statistical heterogeneity was determined by an I^2^ statistic; scores greater than 50% indicate that variability in effect estimates was a result of study heterogeneity rather than sampling error (Higgins, 2008). Associations were calculated using unadjusted logistic regression analysis. Odds ratios (OR) and 95% CIs were reported as measures of effect size and precision of the associations. Analyses were conducted in IBM SPSS version 29 (IBM Corp., 2022), SAS version 9.4 (SAS Institute Inc., 2023), and JBI Sumari (Munn et al., 2019).

## Results

Of the 7,343 people (Australia = 2,703; United Kingdom = 2,243; United States = 2,397) who consented to participate, 6,577 completed the survey (retention rate: Australia = 92.2%; United Kingdom = 87.4%; United States = 88.6%). Participants were then excluded if they indicated that they were either female at birth (Australia = 60; United Kingdom = 47; United States = 92), did not identify as male (Australia = 59; United Kingdom = 42; United States = 77), failed the mid-survey attention check (Australia = 509; United Kingdom = 434; United States = 563), or reported that they had not answered the questions honestly (Australia = 38; United Kingdom = 46; United States = 31; total of 1,591 removed). An additional 68 participants were removed because they were missing data for one or more demographic benchmark variables used for data weighting. This resulted in an analytical sample of 4,918 participants (Australia = 1,939; United Kingdom = 1,506; United States = 1,473). The median survey completion time was 14.65 min (min = 4.43, max = 163.4, interquartile range = 9.52) for the Australian sample, 12.7 min (min = 4.02, max = 84.42, interquartile range = 8.65) for the U.K. sample, and 15.54 min (min = 4.95, max = 151.07, interquartile range = 10.71) for the U.S. sample. No complaints were made about the survey.

### Population Representativeness

Table 3 presents the proportions of ten demographic factors for the unweighted and weighted Australia, U.K., and U.S. study samples, as well as the respective 2021 census male populations. The average absolute difference in proportions between the weighted samples and 2021 census across all categories of each demographic factor were 2.66% (Australia = 3.73%; United Kingdom = 1.00%; United States = 2.81%) for cultural background, 1.18% (Australia = 2.06%; United Kingdom = 0.36%; United States = 1.12%) for marital status, 4.93% (Australia = 3.10%; United Kingdom = 7.70%; United States = 4.00%) for residential location, 1.47% (Australia = 0.50%; United Kingdom = 2.0%; United States = 1.90%) for child in household, and 2.90% (Australia = 3.70%; United Kingdom = 2.8%; United States = 2.20%) for disability or chronic illness. Age, annual household income, educational attainment, and employment status differed by less than 1.0%.

**Table 3.** Proportion (%) of Unweighted and Weighted Demographic Characteristics for the Retained Participants and 2021 Census of the Male Population.

	Australia (*N* = 1,939)			United Kingdom (*N* = 1,506)			United States (*N* = 1,473)		
Demographics	Unweighted (%)	Weighted (%)	Census (%)	Unweighted (%)	Weighted (%)	Census (%)	Unweighted (%)	Weighted (%)	Census (%)
Cultural background (one or more)									
White	82.8	84.3	88.1	88.8	84.0	85.1	78.4	71.0	72.9
Asian	9.0	14.0	17.4	6.0	8.6	9.2	2.8	3.8	7.1
Indigenous	9.2	3.4	3.4	–	–	–	3.0	4.2	2.6
Other	3.9	5.5	13.2	5.2	7.5	6.2	18.8	25.9	30.5
Sexual orientation									
Heterosexual	92.8	91.9	–^a^	92.6	92.7	96.7	92.8	93.5	–^a^
Gay/bisexual/other	7.2	7.3	–^a^	7.4	7.3	3.3	7.2	6.5	–^a^
Marital status									
Married	51.3	44.8	42.7	51.9	51.6	50.8	48.5	49.8	52.1
De facto relationship	16.0	13.8	10.6	17.0	11.1	11.8	9.7	9.7	8.8
Widowed	1.0	1.5	2.3	1.5	3.5	3.4	3.6	2.6	2.7
Divorced/separated	6.6	9.5	10.6	6.8	6.3	6.4	10.6	10.7	11.1
Never been married	25.0	30.7	33.8	22.9	27.5	27.6	27.6	27.3	25.4
Employment status									
Active in workforce	75.5	68.5	68.6	69.6	63.6	65.3	61.8	64.0	64.2
Not participating in workforce	24.5	31.5	31.4	30.4	36.4	34.7	38.2	36.0	35.8
Age									
18–24 years	13.9	15.3	15.3	8.9	11.0	11.0	10.2	12.0	12.2
25–34 years	21.4	17.6	17.6	17.3	17.1	17.1	17.6	18.0	18.0
35–44 years	18.1	16.9	16.9	16.9	16.5	16.5	17.0	17.4	17.4
45–54 years	11.8	15.6	15.6	18.7	17.1	17.1	16.4	16.0	16.0
55–64 years	11.2	14.4	14.4	17.5	16.1	16.1	16.8	16.5	16.5
65+ years	23.5	20.1	20.1	20.6	22.2	22.2	22.1	19.9	19.9
Annual household income									
Low income^b^	18.6	28.9	29.1	17.4	19.0	19.0	15.8	17.4	17.4
Middle income^c^	51.7	46.7	46.6	57.3	69.5	69.5	56.8	48.8	48.7
High income^d^	29.8	24.4	24.4	25.3	11.5	11.5	27.3	33.8	34.0
Highest educational attainment									
Below secondary education	9.6	15.8	15.7	5.8	9.1	9.1	4.7	11.5	11.6
Secondary education	29.8	23.6	23.1	39.3	42.6	42.8	48.5	45.9	45.9
Tertiary education	60.6	60.6	59.5	54.9	48.3	48.5	46.8	42.7	42.7
Residential location									
City or suburb	83.8	82.9	79.8	68.9	70.2	62.5	75.5	77.5	73.5
Rural or regional	16.2	17.1	20.2	31.1	29.8	37.5	24.5	22.5	26.5
Child in household									
One or more	40.2	33.9	33.4	32.5	29.6	31.6	31.5	34.0	32.1
None	59.8	66.1	66.6	67.5	70.4	68.4	68.4	66.0	67.9
Disability or chronic illness									
Yes	21.9	23.2	19.5	21.6	22.8	25.6	32.2	31.3	29.1
No	78.1	76.8	80.5	78.4	77.2	74.4	67.8	68.7	70.9

a Sexual orientation not included in census.

b Less than AUD$50,000 or £20,000 or US$25,000.

c Between AUD$50,000–$149,999 or £20,000–£59,999 or US$25,000–$99,999.

d Equal to or more than AUD$150,000 or £60,000 or US$100,000.

### Prevalence Comparisons

Table 4 presents the proportion of men in the Australia, United Kingdom, United States, and pooled study sample who have sexual interest or offend against children. Accompanying this are the weighted pooled proportions (95% CI; 95% PI) obtained from the extant literature, including pooled sample size, number of unique samples (*k*), and heterogeneity coefficient (*I*^2^) (forest plots available in Supplemental Figures 1–4). The pooled study and meta-analysis samples had similar proportions of men who engaged in online child sex offending (6.0% vs. 5.2%) and were sexually attracted to pubescent children (8.5% vs. 8.8%), while our study had a greater proportion of men who had sexual contact with children offline (4.5% vs. 2.6%) and were sexually attracted to prepubescent children (6.0% vs. 3.1%).

**Table 4.** Proportion (95% CI; 95% PI) of Men Who Sexually Offend or Have Sexual Interest Towards Children.

	Current study				Meta-analysis		
Outcome measure	Australia (*n* = 1,939)	United Kingdom (*n* = 1,506)	United States (*n* = 1,473)	Combined (*N* = 4,918)	Pooled %	Total sample (*k*)	*I* ^2^
Online CSEA	4.8% (95% CI [3.8%, 6.0%])	4.7% (95% CI [3.6%, 6.1%])	9.1% (95% CI [7.5%, 10.9%])	6.0% (95% CI [5.3%, 6.8%])	5.2% (95% CI [2.7%, 8.5%]) (95% PI [2.2%, 12.0%])	16,889 (5)	98.2%
Offline sexual contact	3.2% (95% CI [2.4%, 4.2%])	4.8% (95% CI: 3.7%, 6.2%)	6.0% (95% CI: 4.8%, 7.5%)	4.5% (95% CI [3.9%, 5.2%])	2.6% (95% CI [1.3%, 4.2%]) (95% PI [1.3%, 5.1%])	12,520 (6)	92.5%
Hebephilia	8.7% (95% CI [7.3%, 10.3%])	6.6% (95% CI: 5.3%, 8.2%)	10.3% (95% CI: 8.7%, 12.2%)	8.5% (95% CI [7.7%, 9.5%])	8.8% (95% CI [4.4%, 14.4%]) (95% PI [4.0%, 18.2%])	13,448 (6)	99.0%
Paedophilia	5.1% (95% CI [4.1%, 6.3%])	4.5% (95% CI [3.4%, 6.0%])	8.8% (95% CI [7.3%, 10.6%])	6.0% (95% CI [5.3%, 6.8%])	3.1% (95% CI [1.5%, 5.3%]) (95% PI [1.5%, 6.4%])	21,466 (8)	98.0%

*Note.* Meta-analysis produced from a purposive, non-systematic search of the literature. Proportions are presented for demonstrative purposes only. Studies included in meta-analysis: Ahlers et al. (2011), Alanko et al. (2013), Bártová et al. (2021), Baur et al. (2016), Castellini et al. (2018), Dombert et al. (2016), Mundy and Cioe (2019), Ó Ciardha et al. (2022), Santilla et al. (2015), Seto et al. (2015), and Wurtele et al. (2014). CSEA = child sexual exploitation and abuse.

Within our study, rates of sexual interest and offending against children were higher for the U.S. than the Australian and U.K. samples, a finding consistent with another cross-country comparison included in the meta-analysis (Ó Ciardha et al., 2022). Furthermore, the proportion of U.S. men in our study who engaged in online CSEA (9.1%) or offline sexual behaviours with children (6.0%) was nearly identical to those reported in another study of U.S. men included in the meta-analysis (9.2% and 6.4%; Wurtele et al., 2014).

### Unsure Responses

Table 5 indicates that the highest rates of unsure responses were for items relating to peer engagement in child sexual abuse (range 9.9%–16.0%), followed by if the respondent had ever accidentally watched online child sexual abuse material (CSAM; range 8.6%–11.1%). Unsure responses were least prevalent for questions regarding engagement in sexually explicit webcams with children (range 0.6%–1.0%) or purchasing online sexual interactions or content of a child (range 0.3%–1.0%). Only 1.5% to 2.5% of participants were unsure if they had ever deliberately watched CSAM or had sex or sexual contact with a child, respectively.

**Table 5.** “Unsure” Responses for “Child Sex Abuse” Measures.

Measure/Survey items	Australia (*n* = 1,939)	United Kingdom (*n* = 1,506)	United States (*n* = 1,473)
Peer engagement in online CSEA			
Friend(s) have/suspected of having looked at CSEA	311 (16.0%)	192 (12.8%)	231 (15.7%)
Friend(s) have/suspected of having sent sexually explicit messages to children	267 (13.8%)	162 (10.7%)	215 (14.6%)
Friend(s) have/suspected of having webcammed or livestreamed CSEA	228 (11.8%)	149 (9.9%)	188 (12.8%)
Accidental and deliberate online CSEA viewership			
Accidentally viewed CSEA	216 (11.1%)	143 (9.5%)	126 (8.6%)
Knowingly and deliberately viewed CSEA	48 (2.5%)	24 (1.6%)	22 (1.5%)
Online and offline sexual contact with children			
Flirted or had sexual conversations with a child online	41 (2.1%)	31 (2.1%)	19 (1.3%)
Watched or engaged in a sexually explicit webcam or livestream of a child	20 (1.0%)	12 (0.8%)	9 (0.6%)
Paid for online sexual interactions or content of a child	7 (0.3%)	11 (0.7%)	14 (1.0%)
Had sex or sexual contact with a child	40 (2.2%)	44 (2.9%)	40 (2.7%)
Proclivity for CSEA			
Would watch online CSEA if anonymity was guaranteed and could not be caught	47 (2.4%)	38 (2.5%)	29 (1.9%)

*Note.* CSEA = child sexual exploitation and abuse.

### Participants Response to Survey

The last survey question asked participants if they had any concerns about their sexual interest towards children and would like more information and support; indicating yes to this question redirected participants to a webpage containing the contacts for local and international support services. The proportion of men wanting help was greater for men from the United States (8.8% [95% CI = 7.3%, 10.6%]) than for those from Australia (4.5% [3.5%, 5.7%]) and the United Kingdom (4.3% [3.2%, 5.6%]). The last question asked participants if they had any other comments relating to the survey. Just under half (46.5%) of all participants responded, more than 80% of whom commented some variation of “*no*” or “*thank you*.” Less than 1% of those who commented said that the survey was “*disturbing*,” “*disgusting*,” or “*fucked up*.” By contrast, 5.7% of those who commented said that the survey was “*interesting*,” “*important*,” or “*good*.”

### Age at Offline Sexual Interactions With Children

One-in-twenty men (*n* = 223, 4.5%) indicated that they had sexual contact with someone under the age of 18 while they were over the age of 18 years. Of these men, 43% had engaged in online child sex offending. This figure rose to 67.5% when including men who were sexually attracted to children under 16 years, were concerned about their sexual feelings towards children, would have sexual contact with a child 14 years or younger if they would not be caught, and/or would watch CSAM if they would not be caught. The inclusion of men who also had peers who sexually offend against children online increased this figure to 71.8% (Australia = 75.8%; United Kingdom = 61.1%; United States = 78.4%).

Among men who, as adults, had sexual contact with someone under 18, those without other correlates of child sex offending were 5.18 (95% CI [2.11, 12.66]) times more likely to have engaged in sexual contact with someone under 18 while they themselves were minors, compared to men with other correlates of child sex offending. Men with no other correlates of child sex offending were also significantly more likely to be older (OR = 1.96 [1.56, 2.48]), have a lower annual household income (OR = 1.67 [1.04, 2.69]), and have no children in the household (OR = 3.01 [1.39, 6.53]), but did not significantly differ in terms of marital status, educational attainment, or sexual orientation. They were also significantly less likely to watch violent pornography (OR = 4.20 [1.96, 9.00]) and bestiality (OR = 3.28 [1.44, 7.43]), and have experienced sexual abuse during childhood (OR = 6.88 [2.92, 16.22]).

## Discussion

The “Identifying and understanding child sexual offending behaviours and attitudes in men study” provides a novel opportunity to examine the prevalence and factors associated with sexual interest and behaviours towards children among men from three major English-speaking countries. By incorporating a broad spectrum of demographic, health, and behaviour variables, including internet usage patterns, this study sets a new precedent in the field. Notably, its approach to sampling and data collection addresses a gap in existing literature concerning the paucity of research on undetected child sex offenders in the general population. These findings could significantly improve knowledge on the prevalence and nature of CSEA in the general population. Additionally, the comparison across different jurisdictions offers valuable insights into how cultural, legal, and social contexts influence these behaviours.

The initial estimates produced by this study, indicate the need for policymakers to update and strengthen child protection frameworks, especially online. It highlights the importance of a comprehensive policy approach, incorporating enhanced cyber surveillance, international law coordination, and education to prevent child sexual exploitation. The research also points to the need for considering socio-cultural factors and investing in initiatives to safeguard children online and offline. Future research produced by this study on the patterns and profiles of child sexual offenders could inform the creation of targeted prevention and intervention programmes. Such programmes might include early risk detection, public education, and offender-specific therapeutic interventions. Moreover, the study underscores the value of international collaboration in establishing consistent child protection standards, proposing a shift towards proactive, collective efforts across different sectors to enhance child safety.

This study has several significant strengths, notably its international scope, allowing for comparisons of the characteristics and online habits of men with sexual interest and behaviours towards children across Australia, the United Kingdom, and the United States. This comparative approach addresses a significant gap in the literature by considering how differences in online regulation, surveillance, and socio-cultural contexts might affect offending patterns. Additionally, the comprehensive range of data collected – including demographic details, health issues, social support, and internet use – provides the capacity for greater understanding of the factors associated with child sexual offending.

The findings of this study must be interpreted within the context of its limitations. First, the reliance on self-reported data may introduce social desirability bias, particularly given the sensitive nature of the subject, and despite assurances of anonymity and confidentiality. Second, the initial refusal by one panel company to administer the survey due to its subject matter highlights potential biases in participant recruitment. Although CloudResearch agreed to administer the survey, the refusal by the first company raises questions about whether the topic’s sensitivity might also affect participant willingness and honesty, possibly skewing the sample towards individuals with certain views or experiences. Third, the use of online survey platforms, while practical and cost-effective, may introduce selection bias. The population accessing and choosing to participate in online surveys may not fully represent the broader male population, particularly those with limited internet access or distrust in online research platforms.

Finally, by not distinguishing between the ages of participants at the time of their sexual interactions, the study likely categorised some offline consensual sexual activities (e.g. between a 19 and 17-year-old) as criminal. This could inflate the proportion of men thought to engage in offline child sex offending. Cursory analyses suggest that, among men who had sexual contact with a child during adulthood, those who had no other correlates of child sex offending appeared to significantly differ from those who do in terms of age, income, and other factors associated with paedophilic interests. This raises the probability that the former group engaged in lawful offline sexual interactions. Alternatively, these men could reflect a unique category of offline child sex offender, one that is older, has no childhood history of sexual abuse, is not motivated by sexual feelings or attraction towards children, and engages in no other unlawful behaviour (Wortley & Smallbone, 2014). Nonetheless, a reliable distinction cannot be established within the confines of the data.

Future iterations of this study intend to include samples drawn from non-English and low-income countries, ensuring more diverse and under-researched population are represented. Furthermore, incorporating a longitudinal panel design to track changes over time could offer valuable insights into understanding the impact of age and life-events on patterns of sexual interest and behaviours towards children. Additionally, integrating detailed qualitative interviews into the study could help provide a deeper understanding of the motivations and contexts behind the behaviours studied. Finally, participants will be asked to specify the ages and relationships of children whom they have offended against.

## Supplemental Material

sj-docx-1-jiv-10.1177_08862605251403613 – Supplemental material for The Prevalence and Correlates of Child Sexual Offending Behaviours and Attitudes Among Men in Australia, the United Kingdom, and the United States: Study Methodology Supplemental material, sj-docx-1-jiv-10.1177_08862605251403613 for The Prevalence and Correlates of Child Sexual Offending Behaviours and Attitudes Among Men in Australia, the United Kingdom, and the United States: Study Methodology by Tyson Whitten, Michael Salter and Delanie Woodlock in Journal of Interpersonal Violence

sj-docx-2-jiv-10.1177_08862605251403613 – Supplemental material for The Prevalence and Correlates of Child Sexual Offending Behaviours and Attitudes Among Men in Australia, the United Kingdom, and the United States: Study Methodology Supplemental material, sj-docx-2-jiv-10.1177_08862605251403613 for The Prevalence and Correlates of Child Sexual Offending Behaviours and Attitudes Among Men in Australia, the United Kingdom, and the United States: Study Methodology by Tyson Whitten, Michael Salter and Delanie Woodlock in Journal of Interpersonal Violence

## Notes

1. Weighted by age (see Table 3), annual household income before tax ([a] less than $30,000, [b] $30,000–$49,999, [c] $50,000–$79,999, [d] $80,000–$99,999, [e] $100,000–$149,999, [f] $150,000–$199,999, [g] $200,000 or more), Aboriginal/Torres Strait Islander identity (Yes; No), country of birth (Australia; outside Australia), highest level of educational attainment ([a] completed year 10 or below, [b] completed year 11 or 12 or equivalent, [c] completed cert 3 or 4, or [d] advanced diploma/diploma or university degree), marital status ([a] married or living together, [b] not currently married or living together), workforce participation (see Table 3).

2. Weighted by age (see Table 3), annual household income before tax ([a] less than £20,000, [b] £20,000–£39,999, [c] £40,000–£59,999, [d] £60,000–£99,999, [e] £100,000 or more), ethnic origin ([a] White, [b] Asian, [c] Other), educational attainment ([a] did not complete high school, [b] completed high school or equivalent, [c] vocational degree or apprenticeship, or [d] university degree), marital status ([a] married, [b] living with partner, [c] separated/divorced, [d] widowed, [e] never married), workforce participation (see Table 3).

3. Weighted by age (see Table 3), annual household income before tax ([a] less than $25,000, [b] $25,000–$49,999, [c] $50,000–$74,999, [d] $75,000–$99,999, [e] $100,000–$149,999, [f] $150,000 or more), race ([a] White, [b] Hispanic or Latino origin, [c] African American, [d] Other), educational attainment ([a] did not complete high school, [b] completed high school or equivalent, [c] associates degree, [d] bachelor’s degree or higher), marital status ([a] married, [b] separated/divorced, [c] widowed, [d] never married), workforce participation (as shown).

4. The median weight of each Australian participant was 0.81, with five cases truncated. The median weight for U.K. participants was 0.92, with 18 cases truncated. The median weight for U.S. participants was 0.84, with four cases truncated.

5. Measured in the current study as either deliberately watching online CSEM, sexually interacting with a child over webcam, and purchasing sexual content of a child online.

6. Measured in the current study as having had sex or sexual contact with a person under 18 years of age while over the age of 18 years.
