# Supplementary material for: The Prevalence and Correlates of Child Sexual Offending Behaviours and Attitudes Among Men in Australia, the United Kingdom, and the United States: Study Methodology
Source: J Interpers Violence. 2026 Mar 3;41(7-8):1469–90. doi: 10.1177/08862605251403613 (PMC12960743; doi:10.1177/08862605251403613)
Supplement: sj-docx-1-jiv-10.1177_08862605251403613 – Supplemental material for The Prevalence and Correlates of Child Sexual Offending Behaviours and Attitudes Among Men in Australia, the United Kingdom, and the United States: Study Methodology [file sj-docx-1-jiv-10.1177_08862605251403613.docx]

**
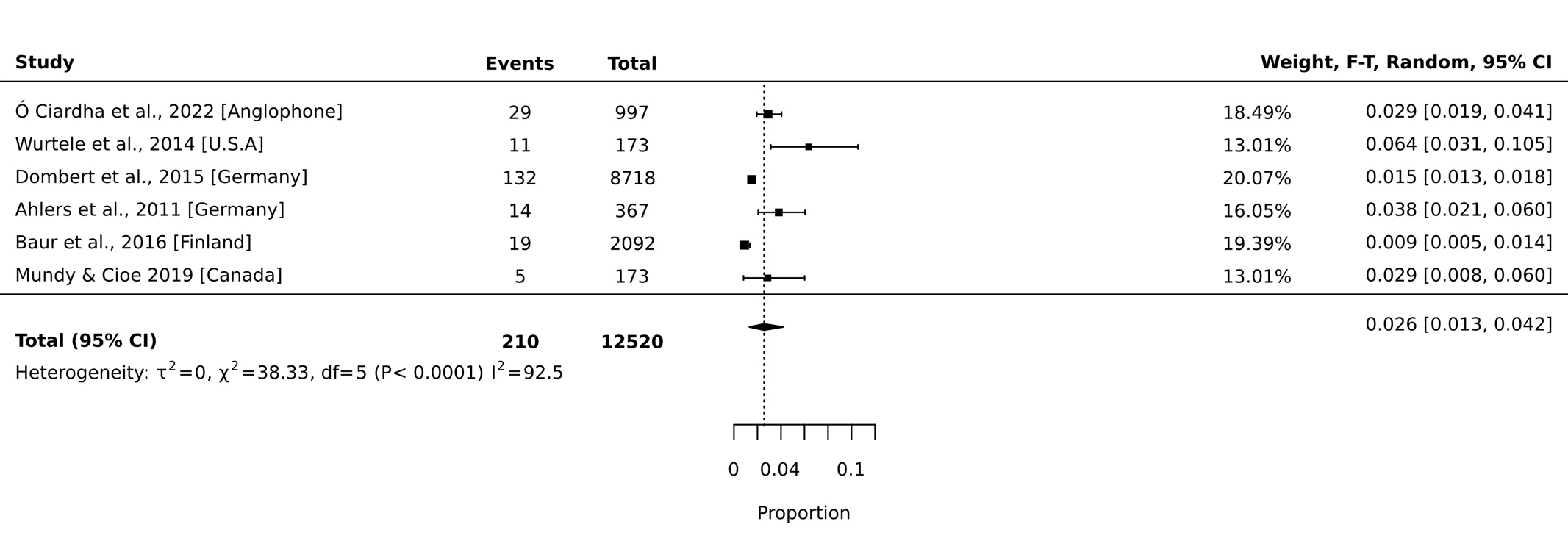
Supplementary Figure S1.** Forest plot of proportion of men who engage in offline sexual contact with children.


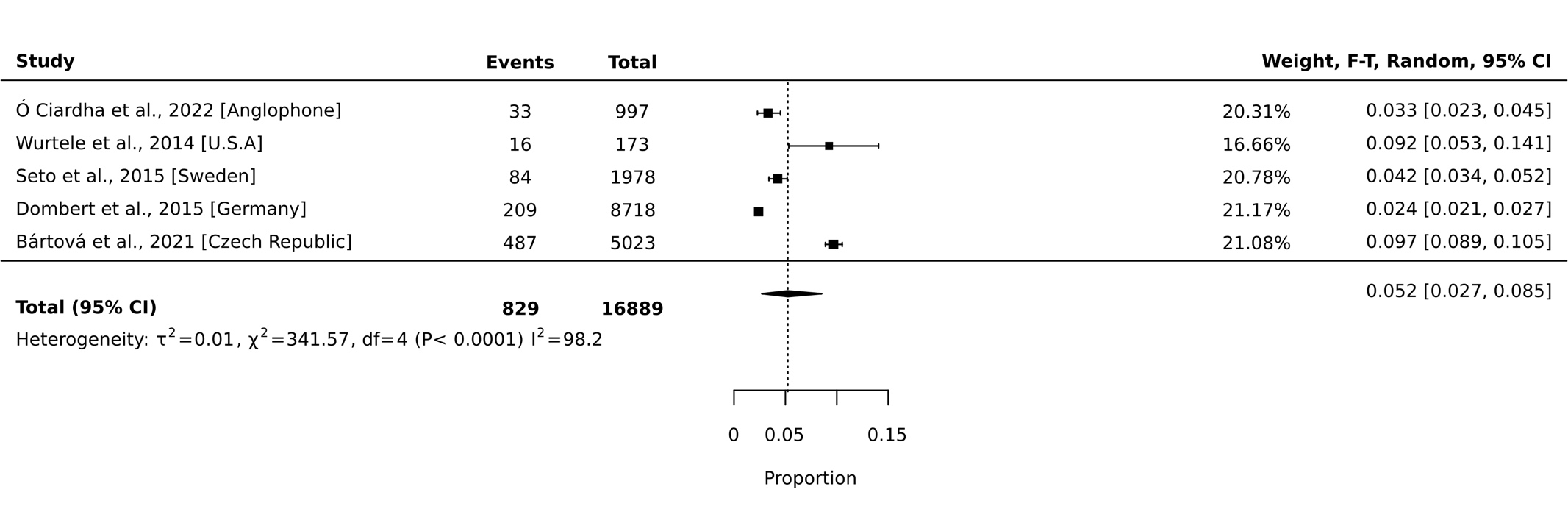
**Supplementary Figure S2.** Forest plot of proportion of men who engage in online CSEA.


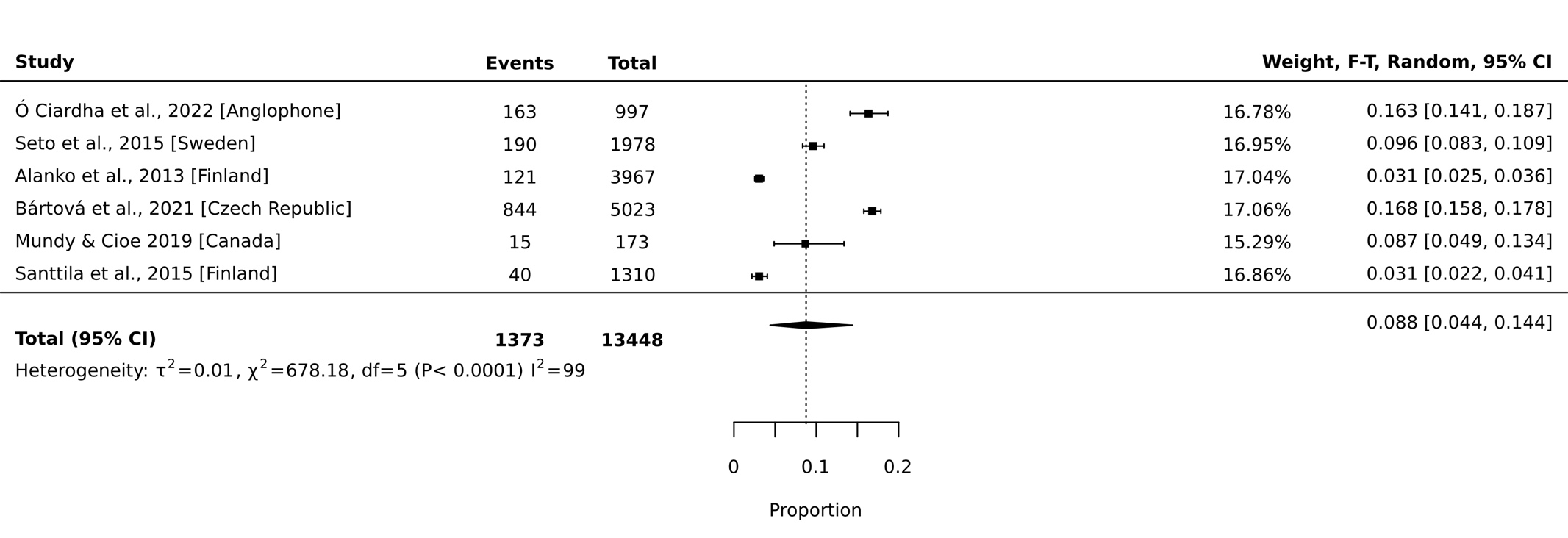
**Supplementary Figure S3.** Forest plot of proportion of men are sexually attracted to pubescent children (hebephilia).


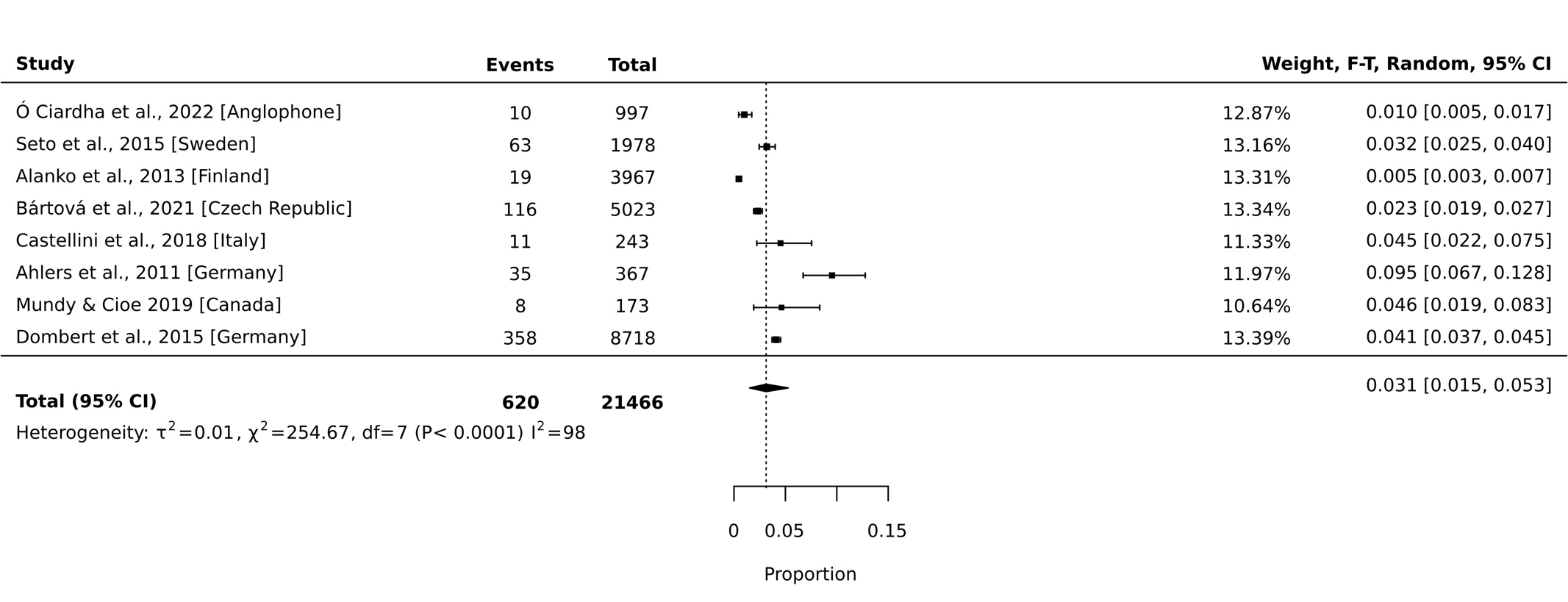


**Supplementary Figure S4.** Forest plot of proportion of men who are sexually attracted to prepubescent children (paedophilia).
