# Supplementary material for: The Prevalence and Correlates of Child Sexual Offending Behaviours and Attitudes Among Men in Australia, the United Kingdom, and the United States: Study Methodology
Source: J Interpers Violence. 2026 Mar 3;41(7-8):1469–90. doi: 10.1177/08862605251403613 (PMC12960743; doi:10.1177/08862605251403613)
Supplement: sj-docx-2-jiv-10.1177_08862605251403613 – Supplemental material for The Prevalence and Correlates of Child Sexual Offending Behaviours and Attitudes Among Men in Australia, the United Kingdom, and the United States: Study Methodology [file sj-docx-2-jiv-10.1177_08862605251403613.docx]

| Supplementary Table S1. Survey items and response categories. | |
| --- | --- |
| **Domain** | **Items** |
| **DEMOGRAPHIC FACTORS** | - **Gender identity of sexual partners** (one or more: Male / Female / Non-binary or other genders / Haven’t had sex / Other (please specify)). - **Sexual orientation** (Heterosexual or straight / Gay / Bisexual / Other (please specify) / Prefer not to say) - **Age** (18-24 years / 25-34 years / 35-44 years / 45-54 years / 55-64 years / 65 years or older) - **Residential location** (City / Suburb / Regional or rural area / Other (please specify)) - **Current marital status** (Married / Living with partner / Widowed / Divorced or separated / Never been married) - **Highest level of educational attainment^*^** (Less than primary school / Primary school / Some Secondary school / Secondary School / Vocational or similar / Some university but no degree / University Bachelor’s Degree / Graduate or professional degree / Prefer not to say) - **Employment status over the last three months** (Full-time / Part-time / Unemployed and looking for work / Homemaker or stay-at-home parent / Student / Retired / Casually worker / Other) - **Total annual household income before taxes^*^** (modified for each country to reflect AUD standardised values: Less than $30,000 / $30,000 - $49,000 / $50,000 - $79,999 / $80,000 - $99,999 / $100,000 - $149,999 / $150,000 - $199,999 / $200,000 or more) - **Cultural background** (Australia only; choose up to two: Non-Indigenous Australian / Indigenous Australian / New Zealander and Pacific Islander / Anglo-European / Non-Anglo European / Asian / Americans / African or Middle Eastern / Unsure / Prefer not to say / Other (please specify)) - **Ethnic origin** (U.K only: White / Black or African or Caribbean / Asian / Mixed / Other (please specify) / Prefer not to say) - **Race** (U.S only; one or more: White or Caucasian / Black or African American / Native American or Alaskan / Asian / Native Hawaiian or other Pacific Islander / Other (please specify) / Prefer not to say) - **Number of children under 18 years living in household** (None / 1 / 2 / 3 / 4 / 5 or more) - **Work currently involves contact with children under** **18 years** (Yes / No) |
| **INTERNET USE** | - **Average hours on a normal business day spent using the internet for work-related purposes** (0 / 1-2 / 3-4 / 5-6 / 7-8 / 9 or more) - **Average hours per day spent using the internet for personal use** (0 / 1-2 / 3-4 / 5-6 / 7-8 / 9 or more) - **Frequency of online activities** (Daily / Weekly / Monthly / Less than monthly / Never / Prefer not to say):   1. Browse or look for information;   2. Send emails;   3. Use social media (e.g., Facebook and Twitter);   4. Use online blogs, forums, or interest groups (e.g., Reddit and Quora);   5. Purchase items from online marketplaces (e.g., eBay and Facebook marketplace);   6. Online banking and other financial activities;   7. Messaging and chatting (e.g., Facebook messenger and Snapchat);   8. Private video chatting over apps and platforms (e.g., Teams and Zoom);   9. Livestreaming videos of self (e.g., YouTube and Instagram Live);   10. Streaming videos on personal device (e.g., Netflix and Amazon prime);   11. Active on romance / dating websites or apps (e.g., Tinder and Hinge);   12. Participate in online gaming or esports;   13. Access sexually explicit websites (e.g., Pornhub and Xvideos). - **Currently use any of the following social media platforms** (Yes / No):   1. YouTube;   2. Instagram;   3. Facebook;   4. Snapchat;   5. Facebook Messenger;   6. Tik Tok;   7. WhatsApp;   8. Twitter;   9. Discord;   10. Skype;   11. Viber;   12. Other (please specify);   13. None . - **Current use any of the following services to prevent tracking and surveillance of online activities** (Yes / No):   1. TOR browser;   2. VPN;   3. Telegram;   4. Signal;   5. WhatsApp;   6. Element;   7. Hive;   8. Private Relay (Safari);   9. Other (please specify);   10. None. - **Currently own any cryptocurrencies** (Yes / No) - **Every used cryptocurrency to purchase items or services online** (Yes / No) |
| **PHYSICAL AND MENTAL HEALTH** | - **Patient Health Questionnaire 4** (Löwe et al., 2010) **– over the past two weeks been bothered by the following problems** (Not at all / 1-7 days / 8-11 days / 12-14 days):   1. Little interest or pleasure in doing things;   2. Feeling down, depressed, or hopeless;   3. Feeling nervous, anxious, or on edge;   4. Not being able to stop or control worrying. - **National Institute on Drug Abuse Quick Screen v1.0** (<https://nida.nih.gov/sites/default/files/pdf/nmassist.pdf>) – **over the past year, how often have used the following** (Never / Once or twice / Monthly / Weekly / Daily or almost daily):   1. Alcohol (5 or more drinks a day);   2. Tobacco products;   3. Prescription drugs for non-medical reasons;   4. Illegal drugs. - **Ever diagnose with a disability or chronic illness** (Yes / No) - **Number of days over the past week unable to carry out usual daily activities fully due to disability or illness** (0 to 7 days) - **Number of days over the past stayed in bed for all or most of the day due to disability or illness** (0 to 7 days) |
| **SOCIAL ADVERSITY** | - **Multidimensional Scale of Perceived Social Supports** (Zimet et al., 1990) (Very strongly disagree / Strongly disagree / Mildly disagree / Neutral / Mildly agree / Strongly agree / Very strongly agree):   1. There is a special person who is around when I am in need;   2. There is a special person with whom I can share my joys and sorrows;   3. My family really tries to help me;   4. I get the emotional help and support I need from my family;   5. I have a special person who is a real source of comfort to me;   6. My friends really try to help me;   7. I can count on my friends when things go wrong;   8. I can talk about my problems with my family;   9. I have friends with whom I can share my joys and sorrows;   10. There is a special person in my life who cares about my feelings;   11. My family is willing to help me make decisions;   12. I can talk about my problems with friends. - **Adverse childhood experiences** (Felitti et al., 1998) (Yes / No):   1. Did a parent or other adult in the household often or very often swear at you, insult you, put you down, or humiliate you, or act in a way that made you afraid that you might be physically hurt?   2. Did a parent or other adult in the household often or very often push, grab, slap, or throw something at you, or ever hit you so hard that you had marks or were injured?   3. Did an adult or person at least five years older than you ever touch or fondle you or have you touch their body in a sexual way, or attempt or have oral, anal, or vaginal intercourse with you?   4. Did you often or very often feel that no one in your family loved you or thought you were important or special, or your family didn’t look out for each other, feel close to each other, or support each other?   5. Did you often or very often feel that you didn’t have enough to eat, had to wear dirty clothes, and had no one to protect you, or your parents were too drunk or high to take care of you or take you to the doctor if you needed it?   6. Were your parents ever separated or divorced?   7. Was your mother or stepmother often or very often pushed, grabbed, slapped, or had something thrown at her, or sometimes, often, or very often kicked, bitten, hit with a fist, or hit with something hard, or ever repeatedly hit for at least a few minutes or threatened with a gun or knife?   8. Did you live with anyone who was a problem drinker or alcoholic or who used street drugs?   9. Was a household member depressed or mentally ill, or did a household member attempt suicide?   10. Did a household member go to prison? |
| **ATTITUDES TOWARDS CSEA** | - **Adapted Child Sexual Abuse Myth Scale** (Collings 1997) (Strongly disagree / Disagree / Neither agree or disagree / Agree / Strongly agree):   1. Sexual images of a person under 18 online where they seem to be happy and enjoying the activity cannot really be described as ‘abusive’;   2. Girls under 18 who share images of themselves nude or in revealing clothing are not to be blamed if an adult responds to them in a sexual way;   3. It’s not harmful to look at nude images of someone under 18 if they took the photo of themselves;   4. Drawn, cartoon or computer-generated sexual imagery of children is wrong;   5. There is nothing wrong with sex dolls that look like children;   6. Viewing a nude or sexual image of a person under 18 is a victimless crime if the person doesn't know that the image was taken;   7. I would still be friends with someone who I knew looked at nude or sexual images of people under 18;   8. People under 18 who act in sexual ways online are not to blame if an adult responds to them in a sexual way;   9. Boys under 18 are sexually experimental and are not harmed when they interact sexually with an adult online;   10. It’s OK to flirt with people under 18 online if you don’t intend to take it further;   11. If a 14 or 15-year-old teenager is on a dating app and contacting adults, they are at least partly responsible if an adult has a sexual interaction with them;   12. People under 18 cannot consent to online sexual interactions with adults;   13. I would not be friends with someone who I knew had sexually interacted online with a person under 18;   14. Online sexual contact with a person under 18 that does not involve actual physical sexual contact or force is unlikely to harm that person psychologically;   15. People under 18 on webcams usually come from poor backgrounds and providing them with money for sexual or nude services is helpful;   16. People under 18 can make their own decisions about how much of their bodies they display on webcam;   17. People under 18 who offer nude or sexual activity on livestream are exploring their sexuality and should not be censored;   18. It is always wrong to pay to view sexual activity with a child on a webcam, even if the child comes from a poor family and their parents need the money;   19. I would still be friends with someone who I knew had webcammed or livestreamed sexually with a person under 18;   20. If someone looks at online sexual images of people under 18 while under the influence of drugs and alcohol, they are still responsible for their actions;   21. Sometimes people look at sexual images or videos of children because they are bored with normal adult pornography;   22. Sometimes people look at sexual images or videos of children because they are very stressed;   23. Some people look at sexual images or videos of children online to prevent themselves from sexually abusing children offline;   24. Viewing sexual images or videos of children is bad only because society says it is;   25. Some people look at sexual images or videos of children because they were abused when they were children. - **Any additional thoughts or beliefs about people who view online sexual content of people under age 18** (Open response) |
| **PORNOGRAPHY VIEWERSHIP** | - **Intentionally watch pornography** (Never watch pornography / Less than once a month / Two to three times a month / Once a week / A few times a week / Daily) - **Ever viewed pornography that included sex with violence or force** (No / Yes, before age 18 / Yes, after age 18 / Unsure) - **Ever accidentally viewed pornography that included sex between humans and animals** (No / Yes, before age 18 / Yes, after age 18 / Unsure) - **Ever knowingly and deliberately viewed pornography that included sex between humans and animals** (No / Yes, before age 18 / Yes, after age 18 / Unsure) - **Ever approached online by an adult offering sexual images, videos, or services** (Yes / No) - **Ever approached online by someone under age 18 years offering sexual images, videos, or services** (Yes / No) - **Ever purchased online sexual services from another person** (Yes / No)   1. Webcam or livestreaming;   2. Subscription service (e.g., Onlyfans or Just For Fans);   3. Nudes or sexual videos from people online;   4. Other (please specify). - **Location of person from whom online sexual services were purchased from^*^** (Host country / Overseas) - **Any additional comments about pornography viewership** (Open response) |
| **CSEA** | - **Have any friends who have or are suspected of having looked at child pornography** (Yes / No / Maybe / Unsure / Other (please specify)) - **Have any friends who have or are suspected of having had sexual conversations online with a person under 18** (Yes / No / Maybe / Unsure / Other (please specify)) - **Have any friends who have or are suspected of having webcammed a person under 18 in a sexual way** (Yes / No / Maybe / Unsure / Other (please specify)) - **Ever accidentally viewed pornographic material containing people under age 18** (No / Yes, before age 18 / Yes, after age 18 / Unsure) - **Ever knowingly and deliberately viewed pornographic material containing people under age 18** (No / Yes, before age 18 / Yes, after age 18 / Unsure) - **Ever flirted or had sexual conversations online with a person under age 18** (Yes / No / Maybe / Unsure / Other (please specify)) - **Ever webcammed in a sexual way with a person under age 18** (Yes / No / Maybe / Unsure / Other (please specify)) - **Ever paid for online sexual interactions, images, or videos involving a person under age 18** (Yes / No / Maybe / Unsure / Other (please specify)) - **Ever had sex or sexual contact with a person below the age 18** (No / Yes, before age 18 / Yes, after age 18 / Unsure) - **If you could be sure that you were anonymous online, would you watch pornographic material containing people below the age of 18** (Yes / No / Maybe / Unsure / Other (please specify)) - **If offered a webcam sex show of a person under age 18, would you watch it** (Yes / No) - **Likelihood you would have sexual contact with a child aged between 12 to 14 years if certain no one would find out and that you would not be punished** (Never / Unlikely / Maybe / Likely / Definitely) - **Likelihood you would have sexual contact with a child aged between 10 to 12 years if certain no one would find out and that you would not be punished** (Never / Unlikely / Maybe / Likely / Definitely) - **Likelihood you would have sexual contact with a child aged under 10 years if certain no one would find out and that you would not be punished** (Never / Unlikely / Maybe / Likely / Definitely) |
| **SEXUAL FEELINGS TOWARDS CHILDREN** | - **Lowest age typically find attractive** (range 0 to 100) - **Highest age typically find attractive** (range 0 to 100) - **Have sexual feelings towards people below age 18** (Yes / No / Maybe / Unsure / Other (please specify)) - **Have concerns about sexual feelings towards people under 18 and would like more information and support** (Yes / No) - **Any additional comments about sexual feelings towards people** (Open response) |
| **EXCLUSION CHECKS** | - **Sex recorded at birth** (Male / Female / Other (please specify) / Prefer not to say) - **Gender Identity** (Man or male / Woman or female / Non-binary / Other) - **When asked to select your favourite colour, you must select 'green'. This is an attention check. What is your favourite colour?** (Red / Blue / Green / Orange / Brown) - **How honest were your responses to the questions asked** (Completely honest / Mostly honest / Somewhat honest / Mostly not honest / Not at all honest) |
| ^*^Categories vary due to inclusion of country specific responses. | |
